# Increasing Vehicular Visible Light Communications Range Based on LED Current Overdriving and Variable Pulse Position Modulation: Concept and Experimental Validation

**DOI:** 10.3390/s23073656

**Published:** 2023-03-31

**Authors:** Cătălin Beguni, Alin-Mihai Căilean, Sebastian-Andrei Avătămăniței, Alin-Dan Potorac, Eduard Zadobrischi, Mihai Dimian

**Affiliations:** 1 Integrated Center for Research, Development and Innovation in Advanced Materials, Nanotechnologies and Distributed Systems for Fabrication and Control, Stefan cel Mare University of Suceava, 720229 Suceava, Romania; 2 Department of Computers, Electronics and Automation, Stefan cel Mare University of Suceava, 720229 Suceava, Romania; 3 Systems Engineering Laboratory of Versailles, University of Versailles Saint-Quentin-en-Yvelines, University of Paris-Saclay, 78140 Vélizy, France

**Keywords:** inter-vehicle communications, LED current overdriving, optical communications, optical wireless communications, traffic safety, vehicle to vehicle communications, visible light communication, VLC range, V2V

## Abstract

Due to its unique advantages, the integration of Visible Light Communications (VLC) in vehicle safety applications has become a major research topic. Nevertheless, as this is an emergent technology, several challenges must be addressed. One of the most important of these challenges is oriented toward increasing vehicular VLC systems’ communication range. In this context, this article proposes a novel approach that provides a significant communication distance enhancement. Different from most existing works on this topic, which are based on refining the VLC receiver, this new article is focused on improving the VLC system based on the benefits that can be achieved through the VLC transmitter. The concept is based on Light-Emitting Diode (LED) current overdriving and a modified Variable Pulse Position Modulation (VPPM). Therefore, LED current overdriving provides the VLC receiver higher instantaneous received optical power and improved Signal-to-Noise Ratio (SNR), whereas the use of the VPPM ensures that the VLC transmitter respects eye regulation norms and offers LED protection against overheating. The concept has been experimentally tested in laboratory conditions. The experimental results confirmed the viability of the concept, showing an increase of the communication range by up to 370%, while maintaining the same overall optical irradiance at the VLC transmitter level. Therefore, this new approach has the potential to enable vehicular VLC ranges that cover the requirements of communication-based vehicle safety applications. To the best of our knowledge, this concept has not been previously exploited in vehicular VLC applications.

## 1. Introduction

New research in the field of developing smart mobility concepts in the smart cities of the future requires finding innovative solutions to ensure communications between both Vehicle-to-Vehicle (V2V) and Vehicle-to-Infrastructure (V2I) on the road, leading in turn to enhanced safety and improved traffic flow [1,2,3,4]. One of the techniques that has gained increasing momentum in recent years is a subfield of Optical Wireless Communications (OWC), namely, Visible Light Communications (VLC) technology [5,6]. This effect comes as a result of the multiple advantages VLC has, such as the transmission of information in a range of unlicensed frequencies, the use of a spectrum about 1000 times greater than the current Radio Frequency (RF) spectrum, the possibility to use the already existing lighting and signaling Light-Emitting Diode (LED) systems with minimal modifications, and last but not least, the compatibility with other technologies that would ensure complementary advantages within hybrid platforms [7,8,9]. Although the performances obtained in recent years are encouraging, with results that a decade ago would have seemed improbable, some challenges still need to be addressed in the context of ensuring specific conditions in the field of road traffic, such as increasing the robustness to noise, the communication range, or further enhancing the mobility [2,5].

To guarantee road safety at high speeds, especially on highways, VLC systems must have the capability to communicate over long distances. This ensures timely prevention or signaling of potential road events. In long-range VLC, the light scattering and atmosphere absorption greatly affect the channel gain, disturbing the transmitted signal [10,11]. Therefore, for longer transmission distances, better receiver sensitivity and higher transmission power are required.

In this context, the present article is focused on extending the range of VLC systems used in the vehicular environment. Dissimilar to most of the existing approaches on this topic, this work proposes a different paradigm, focusing on the enhancements that could be provided by an improved VLC emitter design, rather than strictly orienting on the development of the VLC receiver like most of the studies do [12,13,14,15,16,17,18,19,20,21,22,23,24,25,26,27]. Thus, in order to achieve this purpose, while also focusing on complying with the lighting norms and with the IEEE 802.15.7 specifications [28], this work investigates the possibility of increasing vehicular VLC systems communication range by overdriving the LED transmitter current and by proportionally shortening the duration of the pulses. Thus, this procedure is applied through the use of a modified Variable Pulse Position Modulation (VPPM) approach. Consequently, based on this method, the overall lighting intensity of the device should remain the same, while the instantaneous irradiance should increase almost proportionally, improving in turn the achievable communication range. Therefore, the contributions and merits of this article can be summarized as follows:

➢ It proposes a new concept to improve the communication range of vehicular VLC systems; different from the existing approaches, which are mainly focused on the improvement of VLC receivers, this technique also exploits the benefits that can be provided by the VLC transmitter, further enhancing the communication range;
➢ It proposes an LED current overdrive mechanism compensated by a proportional *ON* time decrease at the VLC transmitter, ensuring higher instantaneous irradiance, while maintaining the overall irradiance within the limits imposed by the standardization norms or by the user requirements;
➢ Different from most existing works that aim to introduce new concepts, this one provides a comprehensive set of experimental results that demonstrate range enhancement of up to 370%;
➢ As far as we know, this is the first article which applies such a technique in vehicular VLC applications.

The rest of this article is organized as follows: Section 2 presents the state-of-the-art approaches for extending the range of a VLC system. Section 3 details the concept of this work. Section 4 presents the hardware and setup design of the VLC experiments. Section 5 offers a discussion based on the results of this article and emphasizes the importance of this work, whereas Section 6 presents the conclusions of this article.

## 2. State-of-the-Art in Long-Range Vehicular Visible Light Communications

Many of the approaches to extend the visible light communication range have as a central element the improvement of VLC receiver performance because the road regulations limit the orientation and maximum values at which lighting and signaling systems can emit in the visible light spectrum. As such, the improvement of VLC transmitters is strictly limited by the regulations issued by the Economic Commission of Europe (ECE) and the Federal Motor Vehicle Safety Standards (FMVSS) of the United States of America (USA), in order to ensure that road illumination is provided by vehicles in good conditions, and without causing glare for other drivers. On the other hand, there is no need to regulate the performance of VLC receivers, as they can be adapted, developed, and improved without legal limitations. In this sense, various techniques used in the architecture of a VLC receiver to increase the range are implemented nowadays, such as: (i) avoiding the saturation of the optical element under the conditions of exposure to parasitic light (for example, direct exposure to sunlight) by limiting the VLC receiver Field of View (FoV) [14,15] or by using optical filters calibrated on the wavelength of the transmitter [16] and/or by implementing various construction techniques of the transimpedance amplifier [15,17], (ii) the use of optical collimators and of aperture averaging [18,19,20], (iii) the selection of more efficient photodetectors, (iv) the use of high-speed Complementary Metal-Oxide-Semiconductor (CMOS)-based VLC receivers [21], (v) the rejection of unwanted signals with the help of band-pass filters or advanced signal processing techniques [12], and (vi) the use of a novel concept of adaptation to the context [22,23].

A combination of all these factors would lead to a better adapted VLC receiver, capable of a long-range VLC link. For example, the real-world experiments in [24] demonstrated a working range of 45 m in highway driving scenarios. A record of a 185 m communication range established with a commercial vehicle headlight in an outdoor scenario was also experimentally proved in [25], while the communication range established with a red traffic light was 188 m in [26], demonstrating that a careful selection of parameters for the VLC receiver could indeed improve the distance of a VLC link, which could be a first step toward the future implementation of a platform adapted to the context of various external disruptive factors. Another approach based on a receiver diversity technology is presented by the authors of [27]. This solution proved the benefit and feasibility of such a system in long-range VLC communications, demonstrating a 100-m outdoor distance, with the Bit Error Rate (BER) below the 7% Forward Error Correction (FEC) limit of 3.8 × 10^−3^. On the other hand, there are relatively few works which also investigate the benefits that could be provided by the VLC transmitter. For example, the authors of [18] investigate the effect of reducing the VLC transmitter emission angle from 120° to 18°, demonstrating a significant increase in communication range. In [29], a custom-made VLC transmitter is used. In this case, in order to improve the communication range, the VLC traffic light radiation pattern is used, having an optimized LED positioning and also a narrow radiation pattern that enable longer communication ranges.

While improvements made on VLC receivers were indeed the logical first steps for long-range performances, some studies also tackled the possibility of overcoming some drawbacks on the emitter side. As such, the authors of [30] provided a novel analytical model where, based on an adaptive energy-saving approach at the VLC emitter level, the saturation of optical receivers caused by parasitic light was mitigated. Another attempt to improve the communication range is through relay-assisted VLC systems, where other vehicles can act like nodes [31,32] in V2V scenarios, or street lamps can act like Access Points (AP) [33] in V2I scenarios, which can also help maintain the communication link in Non-Line-of-Sight (NLoS) conditions. Table 1 provides a summary showing the evolution of vehicular VLC prototypes’ communication range over the time, emphasizing the selected approach for range enhancement. As one can see, the solution proposed in this work is totally different from the previously proposed solutions, and even if the range is not at par with actual development, it should be stressed that current laboratory testing was done with only one cell of three LEDs, resulting in a significantly lower optical irradiance. Future experiments in outdoor condition will follow, integrating real-life vehicular light systems in a complete configuration.

## 3. LED Current Overdriving: Proposed Concept Presentation

**Figure 1:**
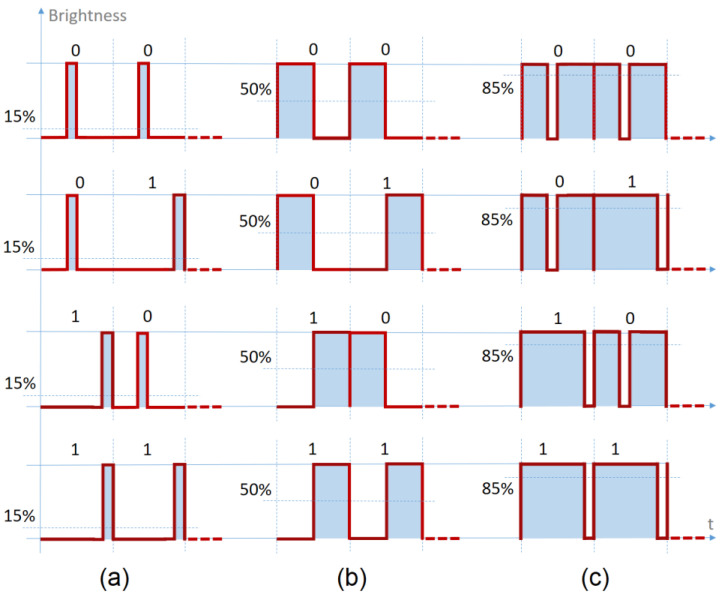
The modified VPPM coding scheme implemented in [34]: (**a**) Average illumination less than 50%; (**b**) Average illumination equal to 50%, the same as Manchester coding; (**c**) Average illumination greater than 50%.

The IEEE 802.15.7 standard [28] states that, for vehicular applications, VLC systems should use On-Off Keying (OOK) modulation with Manchester code and data rates between 11.67 and 100 kb/s. In addition, the standard also mentions for this type of applications, the use of VPPM together with 4B6B coding, providing data rates between 35 and 266 kb/s. In the OOK case, the dimming mechanism is based either on the insertion of compensation symbols, which can significantly decrease the effective data rate, or on the implementation of a VPPM technique, which includes a Pulse Width Modulation (PWM)-based mechanism that enables the control of the pulse width in dependence to the desired duty cycle. One of the main applications of light dimming in vehicles is to use the brake lights system also as a parking light, dimming the light with a duty cycle value of 5–20%. Similarly, this function can be applied to car headlights, using the same illumination system for the low beam as for the high beam, but with a diminished duty cycle. Therefore, to make the dimming function more efficient, the VLC transmitter proposed in [34] uses a particular mechanism of VPPM to control the pulse width according to the desired duty cycle (exemplified as 15%, 50%, and 85% in Figure 1). This is the modified VPPM technique that was also used in the present work.

Experiments from [34] highlighted that the decrease in brightness leads eventually to the reduction of the communication distance. Therefore, it is necessary to find solutions to extend the communication range when dimming is necessary, such as the particular case of switching the car’s lamps between brake and parking lights, while keeping at the same time the necessary illumination for each individual case.

The novel idea in this new work is to increase the communication range by overdriving the LED with an increased value for the forward current, but further decrease the duty cycle rate for restoring the necessary brightness. That would mean that an inevitable chromaticity shift will be present, so a trade-off between this and the advantage of a long-range communication must be taken into consideration.

In order to not exceed the maximum power, an LED must be fed with an average current being under its nominal value. The nominal power $P_{n}$ based on direct forward current $I_{F}$ and direct forward voltage $V_{F}$ is given by Equation (1):

(1) $$P_{n}=I_{F}\cdot V_{F}.$$

Approximating the direct forward voltage $V_{F}$ of an LED as being relatively constant, the value of the pulsed current with a duty cycle D should be similar to the one resulting from Equation (2):

(2) $$I_{F}=I_{P}\cdot T_{i}/T\to I_{P}=I_{F}\cdot T/T_{i}=I_{F}/D,$$

where T is the period, and $T_{i}$ is the interval allocated to the pulse.

For an LED with a direct current $I_{F}=$ 20 mA, applying a duty factor D of 1%, the pulsed current $I_{P}$ could theoretically go up to 2 A. Accordingly, for short intervals of time the light power will increase significantly, allowing a longer communication distance.

If a mono-bit Pulse Position Modulation (PPM) is used, (which is equivalent with Manchester coding), then the interval between two transitions is reduced by half and the peak current can be doubled, as resulting from Equation (3):

(3) $$I_{p_{2}}=I_{d}\cdot \frac{T}{T_{i_{2}}}=I_{d}\cdot \frac{2\cdot T_{i_{2}}}{T_{i_{2}}}=2\cdot I_{d}.$$

**Figure 2:**
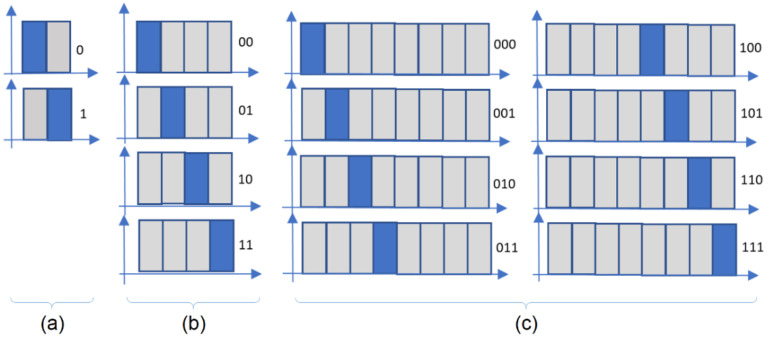
*n*-PPM examples: (**a**) *n* = 2, binary PPM (Manchester code); (**b**) *n* = 4, 4-ary PPM; (**c**) *n* = 8, 8-ary PPM.

Generally, for *n*-ary, *n*-PPM, the distance between transitions is reduced by the factor *n* (Figure 2) [35].

Since the emitting diode is not powered continuously, a multiplied by *n* overdrive current could be used, in accordance with Equation (4):

(4) $$I_{p_{n}}=I_{d}\cdot \frac{T}{T_{i_{n}}}=I_{d}\cdot \frac{n\cdot T_{i_{n}}}{T_{i_{n}}}=n\cdot I_{d}$$

**Figure 3:**
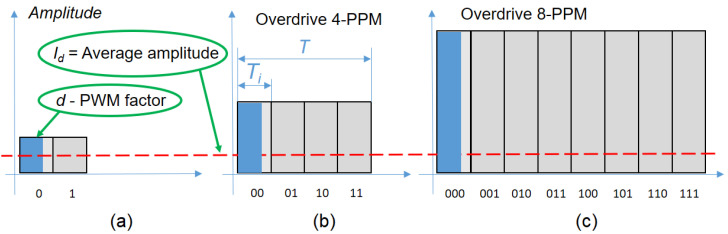
Theoretical analysis for overdriving an LED with a reduced duty factor, using VPPM: (**a**) Manchester code; (**b**) 4-PPM; (**c**) 8-PPM.

If we are to use VPPM (Figure 3), which means to include a Pulse Width Modulation (PWM) in order to further decrease the duty cycle for *n*-PPM, then the overdrive current can be obtained from Equation (5):

(5) $$I_{VPPM_{n}}=I_{d}\cdot \frac{T}{T_{i_{n}}\cdot d}=I_{d}\cdot \frac{n\cdot T_{i_{n}}}{T_{i_{n}}\cdot d}=\frac{n}{d}\cdot I_{d}=\frac{I_{d}}{D}\;,$$

where *d* is the PWM reduction factor. That means that we could further increase the current pulse value for a better performance with a factor of 1/D=n/d, where *D* is the total duty cycle of transmitted message, as previously defined.

Going further with this idea, the concept of an increased amplitude simultaneously with a reduced pulse width could also be applied to the modified VPPM presented in [34]. In this case, the pulse width reduction is based on a Manchester-like coding resulting a modified VPPM, as is illustrated in Figure 1. In this case, the amplitude increase will be inversely proportional with the duty factor decrease as per Equation (5), and this will be the foundation of the present work.

As a side note, multiplying the current will not multiply the luminous output, as luminous efficacy in lumens/watt. In fact, above a certain point, the light output may even decrease as the internal temperature of the LED rises. In any case, for rear brake/parking lights, the cars have red LEDs fitted in the illumination block, and one can safely approximate that the forward current is directly related to the luminous intensity. For a headlight system on the other hand, some necessary correction should be made, because the blue chips present in the white LEDs have different characteristics, which makes the relation between the forward current and the luminous intensity a more non-linear one. In any case, the basic concept idea would remain valid for the white LED as well.

In practice, even if the average current is kept below the nominal value of an LED, we cannot go higher than certain maximum limits. The first concern when driving an LED at the peak of its allowable current is the absolute maximum junction temperature, so proper measures should be taken to dissipate the heat. On these grounds, Equation (6) [36] can be used:

(6) $$T_{J}=T_{A}+R_{TJA}\cdot I_{F}\cdot V_{F},$$

where $T_{J}$ is junction temperature, $T_{A}$ is ambient temperature, $R_{TJA}$ is thermal resistance from junction to ambient and $I_{F}\cdot V_{F}$ is the nominal power drawn by LED, based on forward current $I_{F}$ and forward voltage $V_{F}$. For an already-made design, having the maximum junction temperature $T_{Jmax}$ from the datasheet page, and knowing that the $V_{F}$ will not have very great variations and can be approximated to a constant value, Equation (7) will provide the maximum accepted forward current for a specific ambient temperature:

(7) $$I_{Fmax}=\left(T_{Jmax}-T_{A}\right)/\left(R_{TJA}\cdot V_{F}\right).$$

**Figure 4:**
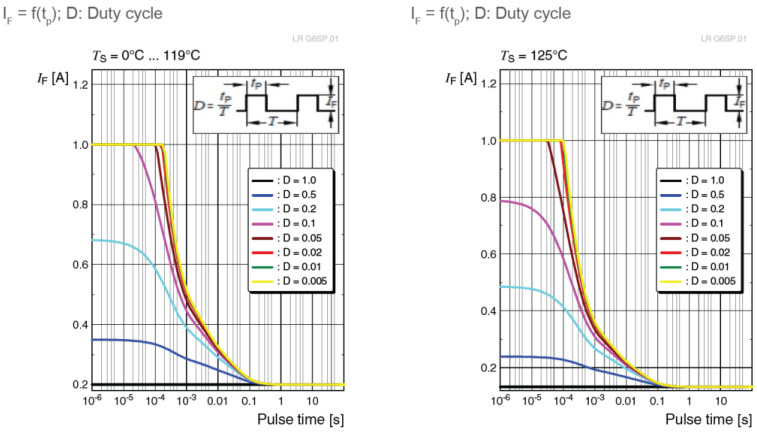
Pulse handling capability of an LR G6SP.01 LED up to 119 °C and for 125 °C [37].

This means that driving the LED with a constant current over the $I_{Fmax}$ value could lead to a potential failure of the emitter. However, driving the LED with a pulsed current would allow this value to be exceeded if some conditions are respected. Usually, the LED producers would indicate in datasheets the minimum duty cycle ratio and the maximum pulse width for a peak forward current. For example, in the case of an LR G6SP.01 made by OSRAM, which supports a continuous forward current of up to 200 mA, the datasheet offers a pulse handling capability for this LED, where the maximum allowable current can go up to 1 A, as shown in Figure 4.

Another aspect that should be taken into consideration is related to the delay of the *ON*/*OFF* switching time. This means that compared to the moment of application of the active front of a direct command (*ON*), the LED will have a delayed increase $t_{ON}$, and, respectively, a decrease also delayed by the $t_{OFF}$ value when the reverse command (*OFF*) is applied. The rise and fall times are limiting the minimum pulse width $T_{pulse\;min}$ in accordance with Equation (8):

(8) $$T_{pulse\;min}=T_{W}+t_{ON}+t_{OFF},$$

where $T_{W}$ is the transmission window, and $t_{ON}$ and $t_{OFF}$ are rise and fall times, defined as the reaching time from 10% to 90% and, respectively, from 90% to 10% of the amplitude. Rise and fall times for power LEDs are typically under 50 ns, depending on the LED’s capacitance. Having a data rate of 10 kb/s up to 100 kb/s with a modified Manchester code means that in this range of 10–100 μs period, the duty cycle can be safely lowered down to 1%, but for higher transfer speeds, reducing the switching times of the LEDs could be necessary, and this can be done by applying over-magnifications to the command fronts.

Over-control may be produced digitally, in one or more stages, in which case the delay of new electronic devices necessary for switching must be considered. A simpler way to accelerate the switching process can be achieved by using derivative circuits based on an accelerating capacitor technique.

## 4. Design and Implementation of the Visible Light Communications System

**Figure 5:**
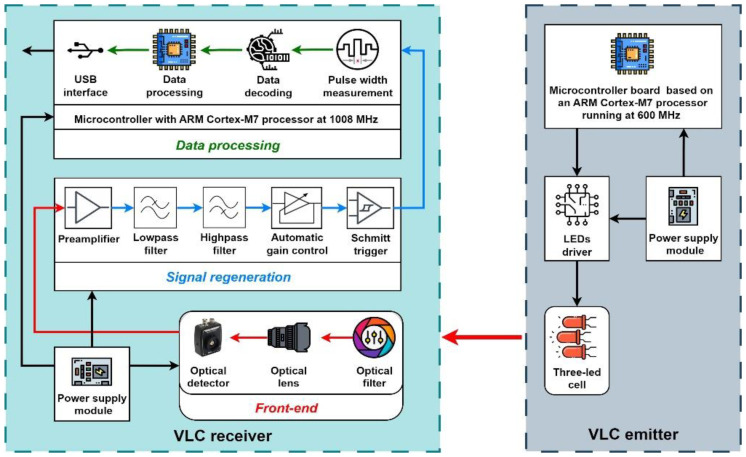
Schematic representation of the VLC prototype system used for the range extension experiments.

**Figure 6:**
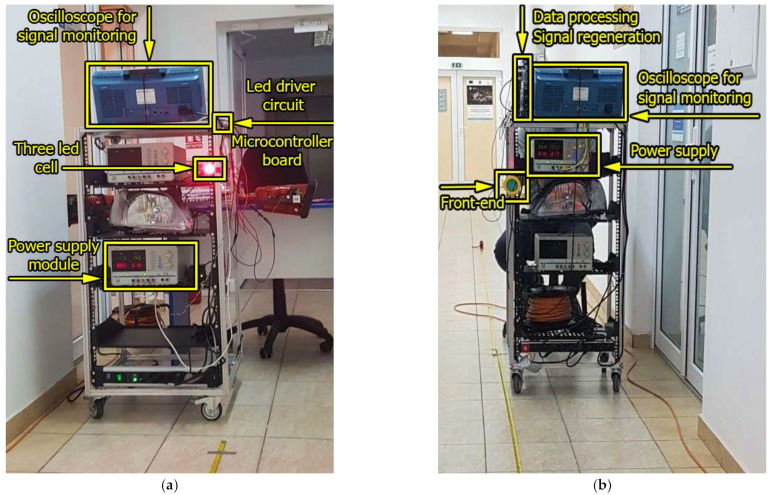
Hardware implementation of the VLC prototype used for the range extension experiments: (**a**) VLC emitter; (**b**) VLC receiver.

To highlight the possibility of extending the communication distance with the help of overdriving an LED, a V2V VLC laboratory prototype similar to the one used in [34] was used, as seen schematically in Figure 5 and pictured in Figure 6. The hardware includes an emitter test bench, with the characteristics presented in Table 2, and a receiver test bench, with the characteristics presented in Table 3. Between these two test benches is the VLC channel, which for the experiments conducted was in laboratory conditions, with natural daylight and artificial light from fluorescent sources.

The central part of the VLC emitter is a 600 MHz microcontroller board based on an ARM Cortex M7 processor, which encodes the data with a particular coding technique previously presented in [34], prepares the necessary frame format with OOK modulation, and directs the information to an LED driver based on a galvanically isolated coreless transformer gate IC made by Infineon. The information is converted into light and sent through the VLC channel with the help of three LR G6SP.01 red LEDs connected in series. This configuration was chosen because in many real-life car taillights driven by LEDs, the structure of various cell formations is made with three LEDs in series in order to efficiently operate under the voltage range of a car battery (12–16 V). For the purpose of exemplification, the braking light was conveniently set at a value of 90 μW/cm^2^. As for the parking light, the recommended value is 5–20% of the intensity light for braking signaling [38], so a value of 6 μW/cm^2^ was chosen for this situation.

The transmitted frames have a synchronization header that signals the start of a new data stream. Some information related to the coding technique, modulation, data rate transfer, and the length of the message follows. At the end of the frame is the payload; this data field has a variable length, specified in the header. The data rate for this experiment was set to 10 kb/s.

The main component of any VLC system is the VLC receiver because its design greatly influences the performance of the whole system; therefore, careful selection of components is needed. The VLC receiver test bench consists of three blocks: the front-end block, the signal regeneration block, and the data processing block.

The front-end starts with an 80 nm band-pass optical filter having the central wavelength of 645 nm, which is suitable for the chosen LEDs. In outdoor conditions, this filter can obstruct up to 80% of optical noise, improving the ability to extract the useful signal. The front-end is also fitted with an optical collecting system that limits the VLC receiver’s FoV. This limitation would improve the Signal-to-Noise Ratio (SNR), but at the same time would impact the mobility, so this challenge should be taken into account in a real-case scenario. The main component of the front-end block is a PIN photodiode connected in a transimpedance circuit, which transforms the electrical current generated by the optical signal into a voltage that can be further processed.

The next block of the VLC receiver regenerates the signal. First, the signal is preamplified, and then it is cleaned up with a band-pass filter. In order to reduce the effect of artificial light sources, the high-pass cutoff frequency was set at 400 Hz, and the effect of high frequency noise sources was eliminated with a low-pass cutoff frequency set at 1 MHz. The signal passes through an Automatic Gain Control (AGC) circuit after that in order to deliver an adequate amplitude signal for the Schmitt trigger circuit, which will output a regenerated digital signal of 5 V amplitude.

The final block takes care of data processing. The regenerated electrical signal is sent to the microcontroller board based on an ARM Cortex M7 processor overclocked at 1.008 GHz, which performs all the data processing in real-time, extracting the information based on the rising and falling edge identification, the pulse width measurement, and the information contained in the received header.

## 5. Experimental Results

### 5.1. Experimental Procedure and Methods

The experimental procedure has the objective to evaluate the effect of overdriving the emitter LED current, while proportionally decreasing the duty cycle, on the communication distance in a vehicular VLC system. The experiments carried on under controlled conditions were done for two situations: one with an average emitted irradiance of around 90 μW/cm^2^, suitable for braking light LED cells, and another one with an average emitted irradiance of around 6 μW/cm^2^, suitable for parking lights. For each situation, the V2V VLC prototype has been tested for various duty cycle values, while the distance between VLC emitter and VLC receiver was gradually increased in 10 cm steps. For the braking lights setup, the prototype was tested with a duty cycle of 50% (Manchester code), 40%, 30%, and 20%, with a step-by-step increased pulse forward current, in order to keep the average current at the same level. The system could not go lower than that, as it would require a current value higher than the 1 A limit imposed by the manufacturer (Figure 4). For the parking light setup, the prototype was tested with a duty cycle lowered in 1% steps, starting from 10%. The LEDs were overdriven with an increased current, in order to maintain the irradiance level set for the parking light. At each step, the BER has been determined in real-time conditions without using any error-correcting protocol. The goal was to have a BER lower than 10^−6^ at each point, with a confidence level of 95%. Because the SNR is relatively high in indoor conditions, the first sign of communication issues was the Loss-of-Frame (LoF) alert, while the BER was still under the established level, so the maximum attainable distance was considered according to the appearance of the LoF alert. It should be mentioned here that the communication distance can still go further than that, at the expense of losing some more frames with every step.

The summary of the testing setup, the parameters of the ambient light, and the equipment used during the experimental evaluation are presented in Table 4 and Table 5.

### 5.2. Experimental Determinations concerning the Effect of an LED Overdrive Pulse Current Combined with a Proportionally Reduced Duty Cycle on the VLC Range

This section presents the experimental results showing the effect of the duty cycle reduction, corroborated with an overdrive pulse current on the V2V VLC link range in two scenarios: braking light and parking light signaling.

#### 5.2.1. Braking Lights Scenario and Results

The laboratory experimental testing followed the route described in Section 5.1. Table 6 summarizes the evaluation results for the braking lights scenario. As can be seen, the pulse forward current was calculated in order to maintain the same average current, which is 175 mA, under the maximum direct forward current indicated by the manufacturer. The irradiance measured at 1 m in ambient darkness shows a good linearity with the average current, so the approximation was correct. The distance attained by lowering the duty cycle but increasing the pulse current in order to keep the average value was higher and higher with each step, and this is the confirmation that a longer range can be obtained with a VLC system when the LEDs are overdriven with a higher pulse forward current. Lowering the duty cycle from 50% to 20% while keeping the same emitted irradiance would more than double the range of communication for a VLC system.

#### 5.2.2. Parking Light Scenario and Results

Table 7 summarizes the evaluation results for the parking lights scenario, which was established at an irradiance of around 6 μW/cm^2^. As expected and already mentioned in Section 3, when the pulse current is at its maximum permissible value (1 A), for a 1% duty cycle, the irradiance is lower than the previous values seen for the same average current, due to the increase in the LED’s internal temperature. However, the attained distance is still greater than the previous step, with a duty cycle of 2%. While at 10% duty cycle and a pulse current of 100 mA, the distance attained was 6.8 m, lowering the duty cycle to 1% and increasing the pulse current to 1 A, the range has more than tripled, achieving an increase of 370%. This confirms that the reduction of the duty cycle leads to an increase in communication distances when a higher pulse current is used for driving the LEDs.

**Figure 7:**
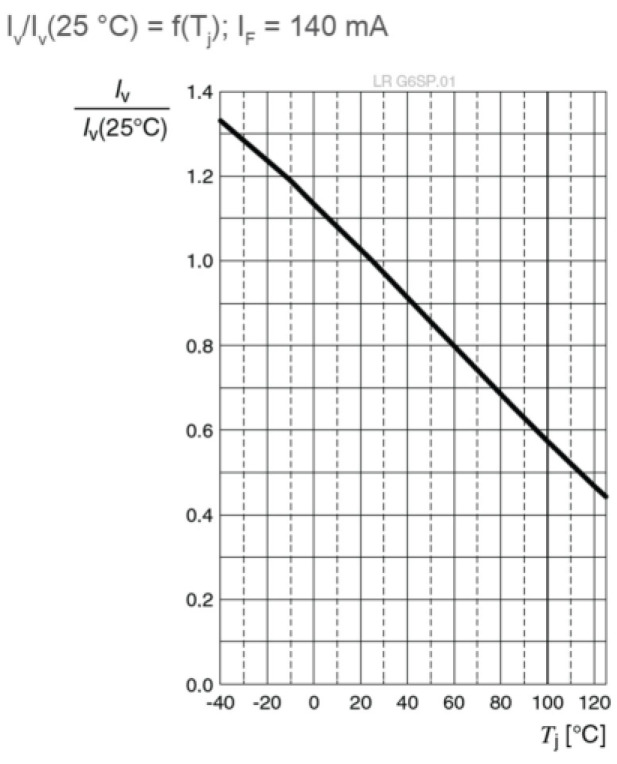
Relative luminous intensity as a function of the junction temperature [37].

Another thing that stands out is the inconsistency between the reaching distance when the same pulse current is flowing through the LED. With a duty cycle of 2% and a pulse current of 500 mA, the reaching distance is 19.1 m, while with a duty cycle of 30% and a pulse current of 583 mA, the reaching distance is only 13.4 m. Again, one of the explanations lies in the relation with the junction temperature. When all the other factors are unchanged, an increase in duty factor would lead to the same increase of the average forward current and, as it can be seen in Equation (6), the junction temperature could be of up to four times higher. In this case, based on Figure 7 extracted from the datasheet, the luminous output would decrease accordingly, leading to a reduction of the maximum range. This contradicts the results obtained in [30], where the distance was decreasing as the duty cycle was lowering for the same pulse current. The explanation was then that the low-pass cut-off frequency chosen at 500 kHz for a better filtration of outdoor noise was affecting the pulses with low duty cycle values, as opposed to the present setup, where the cut-off frequency was adequately chosen at 1 MHz for indoor conditions, where limited noise values are present. Once again, these experiments demonstrate that, for better performance, a trade-off should be taken into consideration between lowering the duty cycle and the quantity of noise allowed in the system while widening the bandwidth of the filter. This confirms the necessity of researching a filter capable of self-adaptation to external conditions. Nevertheless, overdriving the LEDs could increase the maximum range even in outdoor conditions, so further testing will be done in real-life scenarios.

Based on the results presented in Table 6 and Table 7, this study demonstrates that lowering the duty cycle while preserving the same illumination requirement through an increased LED current can lead to a better range in a VLC system. Furthermore, if it is to use this concept in applications that imply higher optical irradiance VLC transmitters, such as traffic lights [26], or vehicle front lights [25], communication ranges of more than 500 m are expected. Consequently, it can be concluded that the method introduced in this article is a major step in overcoming one of the greatest challenges in vehicular VLC applications—the communication range enhancement.

Although not used for the same utilization or purpose and not in the same manner, the idea of a mixed control of duty cycle and of forward current has been previously considered in other applications. Thus, the concept was confirmed to provide better results through a hybrid pulse amplitude and width modulation, as was already highlighted in [39], where the chromaticity shifting was better mitigated with this technique, but also in [40], where higher throughput and improved efficiency was achieved with a new PAM and PWM combination.

On the other hand, although this work was focused on using the proposed concept in vehicular applications, this technique is also suitable for other VLC applications as well. For example, in a context in which 6G technologies are one of the main next challenges for the research community, while the VLC technology is considered as highly promising in this domain [41,42,43], the proposed technique can provide major benefits. Therefore, considering the fact that 6G applications impose high data rates and reduced latencies [41,42,43], the proposed technique can ensure a significant improvement of the SNR, contributing in turn to improved overall performance. Thus, its integration in indoor applications can also facilitate the VLC compatibility with 6G and beyond applications.

## 6. Conclusions

Currently, more solutions are necessary to increase the safety of road traffic, and for situations where RF communications protocols are prone to not having the expected efficiency, such as in heavy traffic scenarios or in tunnels, the VLC can prove to be a life-saving solution as a backup system. One of the most important challenges associated with VLC use in vehicle applications is related to the enhancement of the communication range. Therefore, this article is focused on the possibility of extending the range of these systems in V2V setups. Different from most of the similar works that address this issue, this article focused on the enhancements that could be achieved by optimizing the VLC transmitter. In this context, this article introduced a novel concept which uses LED current overdriving to increase the instantaneous optical irradiance and in turn the communication range, and VPPM to maintain the desired irradiance of the VLC transmitter. The experiments performed in controlled laboratory conditions demonstrated that LED current overdriving and proportional duty cycle decrease generated an increased distance of communication. Therefore, by reducing the duty cycle from 50% to 20% for braking light signaling, the distance was more than doubled, while in the case of parking light signaling, by lowering the duty factor from 10% to 1%, it was possible to increase the communication range by 370%.

The scope of future work is to validate these experiments in real-life scenarios, using commercially available vehicle stop lights, in order to demonstrate the feasibility of this idea in a more complex V2V VLC scenario, while studying at the same time the influence of the noise in outdoor conditions.

## Figures and Tables

**Table 1:** Summary of the vehicular VLC communication range evolution.

Reference	Year	Application Type	Maximum Range [m]	Approach to Improve Communication Range
[14]	2008	I2V	90	Long communication range is achieved based on an extremely narrow 1.3° FoV;
[18]	2012	I2V	31	By adjusting the VLC transmitter emission angle from 120° to 18° and by using a 25 mm focal length lens, communication range is increased from 1 to 31 m;
[29]	2012	I2V	50	Medium communication range provided without totally giving up FoV or robustness to noise; Custom made VLC transmitter with an optimized radiation pattern is used;
[21]	2014	I2V	110	VLC receiver prototype based on a 1000 fps CMOS sensor is used;
[27]	2016	I2V	100	One of the first VLC prototypes able to provide Mb/s data rates and long communication ranges based on a receiver;
[15]	2018	I2V	130	Logarithmic transimpedance circuit and narrow FoV; Custom made VLC transmitter with optical irradiance above standard values;
[16]	2021	V2V	75	One of the longest ranges reported for a standard irradiance vehicle rear light/standard irradiance traffic light/standard irradiance vehicle front light VLC transmitter. The communications range has been achieved based on an improved VLC receiver design;
[26]	2021	I2V	188	
[25]	2021	V2V	185	
This work	2023	V2V; I2V and indoor applications compatible as well	25.2	The communication range is enhanced by up to 370% by increasing the instantaneous optical irradiance, while adjusting the LED on time with the purpose of ensuring the same average optical irradiance and protecting the LED from overheating.

**Table 2:** Parameters of the VLC emitter test bench.

Parameter	Feature/Values
Optical source	3 × LR G6SP.01 (OSRAM red LED)
Emitted irradiance at 1 m distance	≅6 μW/cm^2^ for parking light ≅90 μW/cm^2^ for braking light
VLC emitter’s central wavelength	623 nm
Modulation technique	OOK
Coding technique	Modified VPPM [34]
Data rate	10 kb/s
VLC channel conditions	Indoor condition (laboratory), with natural and artificial fluorescent light and a total irradiance of ≅70 μW/cm^2^
Encoding hardware	Microcontroller board based on an ARM Cortex M7 processor at 600 MHz

**Table 3:** Parameters of the VLC receiver test bench.

VLC Receiver Blocks	Parameter	Values/Features
Front-end	VLC receiver’s FoV	±20° due to the optical collector
	VLC optical photodetector	PDA100A2 switchable gain detector
	Optical filter dominant wavelength	645 ± 40 nm
Signal regeneration	Amplifier’s gain	400, with automatic gain control
	Signal filtering	- 400 Hz high-pass 4th order Bessel filter - 1 MHz low-pass 4th order Bessel filter
	Square signal reconstruction	Schmitt trigger circuit
Data processing	Hardware	Microcontroller board based on an ARM Cortex M7 processor at 1.008 GHz
	Data processing	Based on rising and falling edge identification and pulse width measurement
	Data decoding	Real-time extraction for data modulated using OOK, with data rate of 10 kb/s
	Monitored parameters	Real-time BER computing without forward error correcting codes
Other VLC receiver parameters	Dimming factor	1–40%

**Table 4:** Summary of the experimental parameters.

Parameter	Feature/Values
Testing conditions	High SNR laboratory conditions
Dimming factor	- 20–50% in 10% steps for braking lights - 1–10% in 1% steps for parking lights
VLC emitter	623 nm red LEDs
Emitter–Receiver (V2V) distance	Variable, in 10 cm steps
VLC receiver	PIN Photodiode-based
Ambient light	48 μW/cm^2^
Modulation technique	Modified VPPM as per [34]
Data rate	10 kb/s
Measured parameter	Real-time BER determination without the use of FEC protocols, and LoF alert

**Table 5:** Equipment used during the experiments.

Equipment Type	Model
Irradiance meter	Delta Ohm HD 2302.0 with LP 471 RAD Probe
Multimeter	Fluke 175 true RMS
Optical spectrometer analyzer	Sekonic C-800
Oscilloscope	Tektronix TBS 2104

**Table 6:** Summary of the experimental results showing the viability of the range increasing mechanism for the braking light tests.

Modulation	Data Rate (kB/s)	BER	Conditions	Duty Cycle	Pulse Current (mA)	Emitter Irradiance (μW/cm^2^)	VLC Distance (m)
Modified VPPM	10	<10^−6^	48 μW/cm^2^ ambient light: daylight in indoor conditions with fluorescent light sources on	50%	350	90.2	7.7
				40%	438	89.7	11.3
				30%	583	88.5	13.4
				20%	875	88.4	16.8

**Table 7:** Summary of the experimental results showing the viability of the range increasing mechanism for the parking light setup.

Modulation	Data Rate (kB/s)	BER	Conditions	Duty Cycle	Pulse Current (mA)	Emitter Irradiance (μW/cm^2^)	VLC Distance (m)
Modified VPPM	10	<10^−6^	48 μW/cm^2^ ambient light: daylight in indoor conditions with fluorescent light sources on	10%	100	5.65	6.8
				9%	111	5.61	7.3
				8%	125	5.75	7.9
				7%	143	5.98	8.9
				6%	167	6.01	9.9
				5%	200	5.96	11.0
				4%	250	5.87	13.0
				3%	333	5.82	15.3
				2%	500	5.81	19.1
				1%	1000	5.3	25.2

## Data Availability

Not applicable.
